# Role of the Metal Center in the Modulation of the Aggregation Process of Amyloid Model Systems by Square Planar Complexes Bearing 2-(2′-pyridyl)benzimidazole Ligands

**DOI:** 10.3390/ph12040154

**Published:** 2019-10-12

**Authors:** Daniele Florio, Ilaria Iacobucci, Giarita Ferraro, Ahmed M. Mansour, Giancarlo Morelli, Maria Monti, Antonello Merlino, Daniela Marasco

**Affiliations:** 1 Department of Pharmacy, University of Naples Federico II, 80134 Napoli, Italy; floriodaniele1@gmail.com (D.F.); giancarlo.morelli@unina.it (G.M.); 2 Department of Chemical Sciences, University of Naples Federico II, 80126 Napoli, Italy; ilaria.iacobucci@unina.it (I.I.); montimar@unina.it (M.M.); 3 CEINGE Biotecnologie Avanzate S.c.a r.l., University of Naples Federico II, 80145 Napoli, Italy; 4 Department of Chemistry Ugo Schiff, University of Florence, 50019 Sesto Fiorentino (FI), Italy; giarita.ferraro@gmail.com; 5 Department of Chemistry, Faculty of Science, University of Cairo, Gamma street, Giza 12613, Egypt; mansour@sci.cu.edu.eg

**Keywords:** amyloid aggregation, metallodrugs, gold(III) compounds, platinum(II) compounds, palladium(II) compounds, anti-aggregation properties

## Abstract

The effect of analogue Pd(II)-, Pt(II)-, and Au(III) compounds featuring 2-(2′-pyridyl)benzimidazole on the aggregation propensity of amyloid-like peptides derived from Aβ and from the C-terminal domain of nucleophosmin 1 was investigated. Kinetic profiles of aggregation were evaluated using thioflavin binding assays, whereas the interactions of the compounds with the peptides were studied by UV-Vis absorption spectroscopy and electrospray ionization mass spectrometry. The results indicate that the compounds modulate the aggregation of the investigated peptides using different mechanisms, suggesting that the reactivity of the metal center and the physicochemical properties of the metals (rather than those of the ligands and the geometry of the metal compounds) play a crucial role in determining the anti-aggregation properties.

## 1. Introduction

The research field involving the use of metal-based drugs as inhibitors of amyloid fibril formation and toxicity, targeting neurodegenerative diseases like Alzheimer’s (AD) and Parkinson’s Disease (PD), is experiencing a great flowering [1,2]. In general, transition metal complexes have tunable properties, depending on the oxidation and spin states of the metal center as well as the coordination geometry. All these features could influence the reactivity of these compounds with amyloidogenic species and consequently modulation of their aggregation pathways [3,4].

Metal complexes of relatively kinetically inert metal ions (such as Pt(II) square-planar complexes) can form stable coordinate bonds with amyloidogenic peptides in their monomeric state [5]. Selective modulation of self-recognition mechanisms was obtained by using Pt compounds bearing hydrophobic phenanthroline(phen)-based bidentate ligands and two monodentate ligands (e.g., chlorides) [5]. These molecules bind Aβ_1–40_ via the exchange of the monodentate ligands followed by coordination of the Pt(II) center to the imidazole of His residues [6]. The metal complexes target the N-terminal domain of Aβ through π-π interactions between the phen ligand and the aromatic residues of the peptide (Phe^4^, Tyr^10^, and Phe^19^). (Pt(II)(phen)–Aβ)^2+^ adducts, formed upon the coordination of the metal center to Aβ residues, inhibit peptide aggregation by causing the precipitation of amorphous aggregates rather than the formation of amyloid fibrils. The (Pt(II)(phen)–Aβ)^2+^ adducts also limit the neurotoxicity of Aβ and protect against Aβ-induced synaptotoxicity in mouse hippocampal tissue [6]. The aromatic interactions cause an acceleration in the rate of substitution of ligands of the metal center as demonstrated by results obtained with Aβ_1–16_, a shorter variant of Aβ_1–40_ [7]. Similar results have been obtained using a water-soluble sulfonated analogue, (PtCl_2_(4,7-diphenyl-(1,10)phenanthroline disulfonate)), providing a mixture of products primarily involving the coordination to the sulfur atom of Met^35^ [8], while the reaction of cisplatin does not affect any of the biochemical and cellular effects of Aβ_1–40_ [6].

Direct coordination of the Pt center to amyloid peptides/proteins is not the only strategy that has been identified to inhibit the formation of amyloid fibers. It has been shown that metal-driven oxidation of crucial residues could be also exploited [4]. Indeed, the oxidation of Met^35^ affects the aggregation of Aβ_1–40_ [9]: indeed, when Met^35^ forms the hydrophilic Met sulfoxide, the electronic properties of Aβ peptides vary and cause an enhancement of peptide’s polarity, which hampers hydrophobic interactions that are crucial for the initiation and progression of Aβ aggregation. Thus, it is possible to trigger the oxidation of Met in amyloid peptides utilizing metal compounds that can act as reducing agents or that can produce reactive oxygen species (ROS) which can be responsible for the oxidation [10].

Beyond Pt(II) complexes, other metal complexes have been used as inhibitors of amyloid fiber formation. Due to the ligand field similarities between Pd(II), Au(III), and Pt(II) compounds, the ability of Pd(II) and Au(III) compound to inhibit the fiber formation has been investigated [11,12,13,14,15]. Pd(II) compounds can exchange ligands 10^5^ times faster than their Pt(II) analogues [16], enhancing their capability to interact with the cellular components such as sulfur-donor biomolecules. To overcome this issue, chelating ligands were used to increase the thermodynamic stability of these metal compounds [17,18]. Chelating ligands have been also used to increase the stability of Au(III) complexes in aqueous solvents [19]. Pd(II) complexes containing chelate ligands bind prion protein (PrP) fragments with high affinity and site selectivity. In particular, these molecules can bind the peptide 106–126 of the prion protein (PrP_106–126_) close to the imidazole of a His residue that has a crucial role for aggregation [20,21] or close to amide nitrogen atoms of the peptide, significantly inhibiting the peptide aggregation propensity [15]. Gold-sulfur complexes bind PrP_106–126_ [13] and the human islet amyloid peptide [12] via metal coordination and hydrophobic interactions.

**Figure 1:**
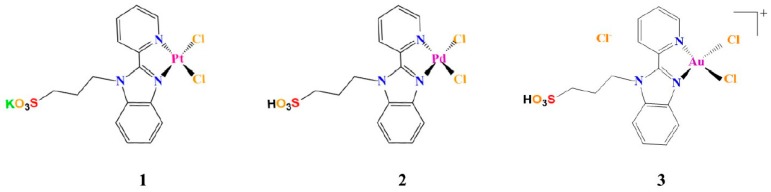
Chemical structures of square-planar complexes investigated in this study.

Here we report on the ability of Pd(II), Pt(II), and Au(III) complexes, bearing 2-(2′-pyridyl)benzimidazole as a ligand (Figure 1), to modulate amyloid-like aggregation of two model amyloid peptides. The complexes bearing the pyridylbenzimidazole skeleton with sulfonate ligands form different types of DNA-complex adducts [22]. They exhibit high cytotoxicity against non-cancerous cells such as HEK293 human embryonic kidney cells [22,23,24]. They are able to bind the model proteins hen egg white lysozyme and bovine pancreatic ribonuclease, as already reported for similar complexes [25,26], both non-covalently and via coordination to side chains of protein residues [27].

Peptide sequences chosen as a model of amyloids are reported in Table 1. They are: (i) the fragment corresponding to the 2nd helix (residues 264–277) of the C-terminal domain of nucleophosmin 1 (NPM1_264–277_), which is able to form amyloid-like assemblies and fibrils toxic to neuroblastoma cells [28,29,30,31,32,33] and (ii) the fragment spanning residues 21–40 of the beta-amyloid peptide Aβ (Aβ_21–40_) [34].

Chosen protein fragments demonstrated directly involved in the aggregation mechanism of the related proteins, NPM1c+ [35] and Aβ_1–42_ [36].

## 2. Materials and Methods

### 2.1. Peptide and Metal Compound Synthesis

Peptide sequences analyzed in this study were synthesized as previously reported [37], in acetylated and amidated version and are reported in Table 1. Reagents for chemical synthesis were obtained from Iris Biotech (Marktredwitz, Germany) and solvents were purchased from Romil (Dublin, Ireland). Peptides were treated with HFIP (at 50% (v/v) in water), purified through RP-HPLC and then identified through LC-MS system LCQ DECA XP Ion Trap mass spectrometer from ThermoFisher (Waltham, MA, USA). Purified peptides were lyophilized and stored at −20 °C until use. A small aliquot of Aβ_21–40_ was oxidized at 1 mg/mL in phosphate buffer (100 mM, pH = 7.2, H_2_O_2_ 0.1% (v/v)), under stirring for 20 h and then further purified [38].

The metal compounds (Figure 1) were synthetized as previously described [22,39].

### 2.2. Interaction of the Metal Compounds with Amyloid Peptides

#### 2.2.1. UV-Vis Absorption Spectroscopy

The reactivity of **1**–**3** towards amyloid peptides was spectrophotometrically investigated at 25 °C in 10 mM borate buffer at pH = 9.0 for NPM1_264–277_ and 10 mM sodium phosphate buffer at pH = 7.4 for Aβ_21–40._ The final concentration of DMSO (Merck KGaA, Darmstadt, Germany) in these solutions was < 0.5%. The electronic absorption titration was carried out at a fixed concentration of the compounds (40 µM), gradually increasing the concentration of peptides by adding 1.0 µL of 2 mM stock solutions in water, kept at 0 °C. Following this addition, the solutions were stirred for 5 min and the spectrum was recorded. These conditions ensured a prevalent monomeric state of added peptides allowing to evaluate each addition in terms of equivalents to each metal complex. Spectra are reported in the ranges 240–500 and 280–500 nm for Aβ_21–40_ and NPM1_264–277_, respectively.

In control experiments, the stability of compounds under the investigated experimental conditions were tested using UV-Vis absorption spectroscopy (see Figure S1 and Supporting info for further details). UV-Vis absorption spectra of **1**–**3** as a function of time were recorded over 24 h on a Varian Cary 5000 UV-Vis-NIR spectrophotometer at 25 °C in 10 mM sodium phosphate buffer at pH 7.4 and 10 mM borate buffer pH at 9.0. Other experimental settings were wavelength range: 240–700 nm, scan-time: 600 nm/min, band width: 2.0 nm, data pitch: 1.0 nm. The three compounds have been dissolved in 100% DMSO and then added to the selected buffers at a final concentration of 5 × 10^−5^ M. The final concentration of DMSO is < 0.5%. The stability of complexes was also evaluated in 10 mM ammonium acetate buffer at pH 6.8 that was employed for mass samples (Figure S1), see below. Since NPM1_264–277,_ has an intrinsic absorption band at 275 nm due to the presence of a Tyr residue_,_ an additional control experiment was carried out to assess that observed signal variations are due to an effective interaction of the metal compounds with the peptide (Figure S2) [22,39].

#### 2.2.2. ESI-MS Analyses of the Adducts among Metal Complexes and Amyloid Peptides

Solutions of NPM1_264–277_ and Aβ_21–40_ at a concentration of 100 µM and 50 µM respectively were incubated in 15 mM ammonium acetate at pH 6.8 with a 1:10 peptide:complex molar ratio, at 25 °C. Incubation times were fixed to 0, 3, and 17 h and analyzed by using a Q-ToF Premier (Waters, Milford, MA, USA) by direct injection at 10 µL/min flow rate. The source parameters were: 3.7 kV for capillary voltage and 42 kV for cone voltage. The acquisition range was set from 600 to 2500 m/z for NPM1_264–277_ and from 700 to 2500 m/z for Aβ_21–40_. Raw data were elaborated by using MassLynx 4.1 software (Waters, Milford, MA, USA). Peptides in the absence of the metal complexes were analyzed as controls (see Figures S3 and S4 in Supporting info).

### 2.3. Fluorescence Assays

To study the aggregation propensity of the peptides in the absence and in the presence of the metal complexes, thioflavin (ThT) fluorescence time-course experiments were carried out at 25 °C using a Jasco FP 8300 spectrofluorometer (JASCO, Tokyo, Japan). λ_exc:_ was set to 440 nm, while λ_em_ was set to 483 nm. ThT concentration was 50 µM, peptide concentration was 100 µM. The solution was maintained under magnetic stirring in a 10 mm path-length quartz cuvette. Experimental conditions: 0.1% DMSO, 10 mM borate buffer (Merck KGaA, Darmstadt, Germany) at pH = 9.0 for NPM1_264–277_ and 0.1% DMSO, 10 mM sodium phosphate buffer for Aβ_21–40_. Reported fluorescence values are the mean of two independent experiments.

## 3. Results and Discussion

### 3.1. The Aggregation Propensity of Amyloidogenic Peptides is Affected by the Presence of **1**, **2**, and **3**

**Figure 2:**
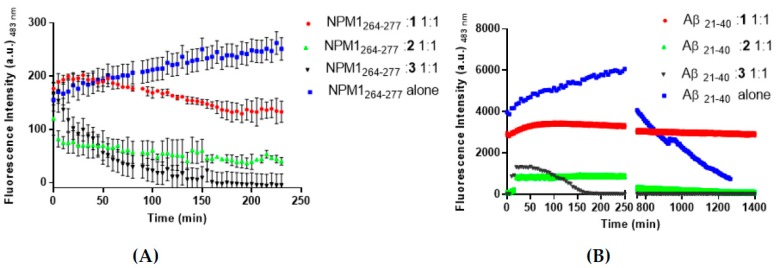
Time-course of ThT fluorescence emission intensity of NPM1_264–277_ (panel **A**) and Aβ_21–40_ (panel **B**) in the presence of **1**–**3**, upon incubation at 1:1 peptide to metal compound molar ratio. The peptides alone are reported as blue squares. Results are representative of two independent experiments.

The ability of **1**–**3** to affect the aggregation process of NPM1_264–277_ and Aβ_21–40_ was monitored through time-course ThT fluorescence emission. For both peptides, the no-zero starting values of ThT fluorescence is due to a fast aggregation during sample preparation, as already observed [5,28]. The presence of the metal complex causes a decrease of ThT signal when compared to the signal of the peptides alone, suggesting an inhibitory effect on amyloid self-aggregation. Different starting intensities suggest that quenching/direct binding mechanisms between metal complexes and ThT can occur. In detail, when NPM1_264–277_ is stirred with **1**, after 50 min, a slow reduction of the fluorescence signal is observed. At the end of the experiment, the ThT fluorescence value presents an intensity comparable with the starting value of the peptide alone (Figure 2A). A more significant decrease in the emission intensity was observed when the ThT fluorescence of the peptide was recorded in the presence of **2**. In this case, a lower signal with respect to t = 0 is attained after 5 min, while after 230 min the fluorescence intensity is very low. The ThT fluorescence signal of the peptide is also significantly affected by the presence of 3. After a steady emission of 35 min, a significant reduction of the emission, at values comparable to those shown in the presence of **2**, is observed.

Similar results were obtained with Aβ_21–40_ peptide (Figure 2B), indeed both **1** and **2** provided a slow signal decrease, delayed with respect to NPM1_264–277,_ while the presence of **3** seems to have a faster effect on the aggregation process.

### 3.2. **1** and **2** Are Able to Disaggregate Amyloid Assemblies

**Figure 3:**
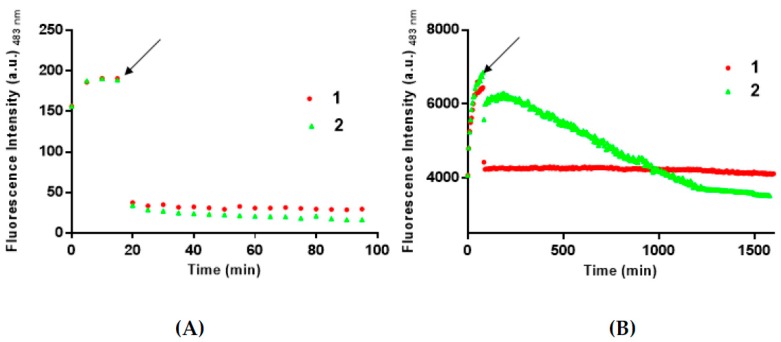
Time-course of ThT fluorescence emission intensity of NPM1_264–277_ (panel **A**) and Aβ_21–40_ (panel **B**) upon addition of 1 (red circle) and 2 (green triangle). The addition is indicated by an arrow. Results are representative of two independent experiments.

The ability of **1** and **2** to disaggregate soluble amyloid aggregates were then evaluated by monitoring the ThT signals versus time upon the addition of the metal complexes to NPM1_264–277_ and Aβ_21–40_ aggregates (Figure 3). As already reported [5], the two peptides have rather different aggregation times, since they differ both in amino acidic composition and length. Thus, **1** and **2** were added to the aggregates at different times, as indicated in Figure 3 (after 15 min for NPM1_264–277_ and after 90 min for Aβ_21–40_).

For NPM1_264–277_, a fast decrease of ThT fluorescence intensity is observed upon the addition of both **1** and **2** (Figure 3A). For Aβ_21–40_ (Figure 3B), a fast decrease of ThT fluorescence intensity is monitored upon the addition of **1**, while a slower decrease of fluorescence signal was observed in the presence of **2**.

Altogether the results of ThT experiments suggest that the three metal complexes could be able to module the aggregation process and that **1** and **2** could also act as disaggregating agents. The results obtained on these last experiments also suggest different kinetics of the recognition processes of the oligomeric form of Aβ_21–40_ by **1** and **2**.

### 3.3. The Ligand Fields of **1** and **2** Are Tuned by the Presence of Amyloidogenic Peptides

**Figure 4:**
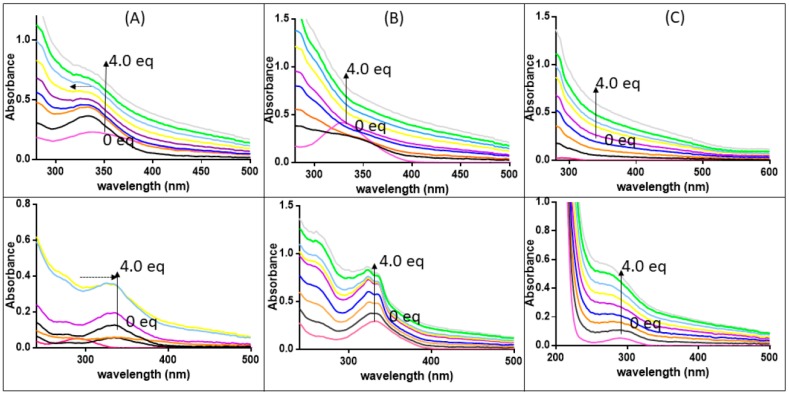
Absorption spectra of **1** (panel **A**), **2** (panel **B**), and **3** (panel **C**) upon the addition of NPM1_264–277_ (top) and Aβ_21–40_ (bottom) at different equivalents. (0 eq fuchsia, 0.5 eq black, 1.0 eq orange, 1.5 eq blue, 2.0 eq violet, 2.5 eq yellow, 3.0 eq light blue, 3.5 eq green, 4.0 eq grey) Vertical arrows indicate changes in intensity, horizontal arrows indicate shifts of λ_max._

To understand at the molecular level how the presence of the three compounds can alter the aggregation (and in the case of **1** and **2** the disaggregation) process of the investigated amyloidogenic peptides, spectroscopic and spectrometric studies were carried out. First, the effects of the presence of NPM1_264–277_ and Aβ_21–40_ on the spectroscopic properties of **1**–**3** were investigated. In particular, the changes of the intensity and/or of the position of Ligand to Metal or Metal to Ligand Charge Transfer (LMCT or MLCT) bands in the UV-Vis absorption spectra [22,39], upon the addition of peptide to a solution of the metal compounds at a fixed concentration, were monitored. The addition of both amyloid sequences to **1** (Figure 4A) causes slightly shifts of λ_max_ that is hypsochromic for NPM1_264–277_ (at 335 → 333 nm) and bathochromic for Aβ_21–40,_ (290 → 330 nm)_._ These features together with the changes in the absorption of the bands suggest a possible modification in the ligand field of Pt, which could be associated with the presence of potential Pt ligands in the sequence of the peptides (the side chains of residues C, M, E, D, N, K, and R). Amyloid peptides affect the absorption spectra also in the case of the Pd compound (Figure 4B). In particular, spectra of 2 recorded in presence of Aβ_21–40_ clearly present a progression in a hyperfine resolution of the initial band centered at 332 nm. The same experiments carried out for **3** (Figure 4C) do not show significant variations of signals.

### 3.4. **1** and **2** Form Adducts with NPM1_264–277_

More detailed information on the interaction between the peptides and the metal complexes were obtained by electrospray ionization mass spectrometry (ESI-MS) (Waters, Milford, MA, USA) experiments. ESI-MS analyses of NPM1_264–277_ and Aβ_21–40_ in the presence of the metal complexes were carried out in a time-dependent way: spectra have been acquired after 0, 3 and 17 h of incubation of each peptide with **1**–**3**.

ESI-MS spectra of NPM1_264–277_ suggest that the solution of the peptide has a heterogeneous composition, due to the presence of a reactive cysteine. Indeed, in addition to the expected species with an MW of 1771.20 Da, a dimeric form of NPM1_264–277_ stabilized by a disulfide bridge (3540.63 Da) is also present, as well as a species showing a molecular weight of 1874.09 Da (Figure S3A). In addition, up to three oxidized species of the NPM1_264–277_ monomer are present. Under reducing conditions, the monomeric form of NPM1_264–277_ is the only detected species (Figure S3B).

ESI-MS spectra of the peptide in the presence of the three metal complexes indicate that **1** and **2** form adducts with NPM1_264–277,_ whereas **3** does not seem to interact with the peptide.

**Figure 5:**
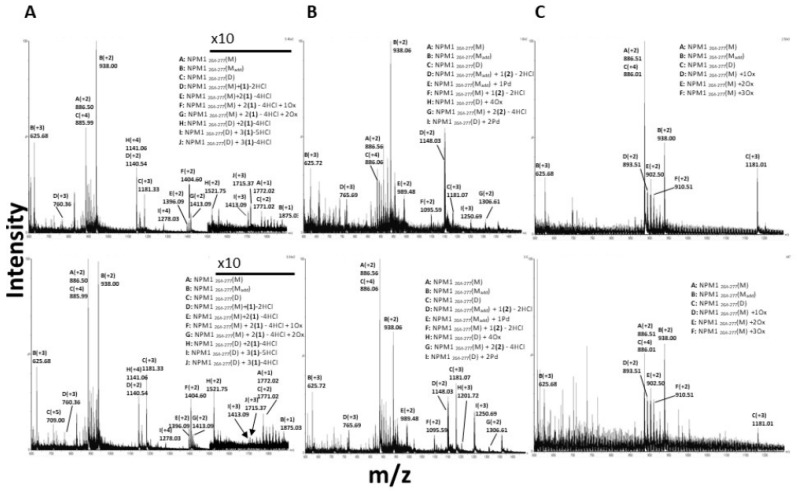
ESI-MS spectra of NPM1_264–277_ incubated with: (**A**) **1**, at t = 0 (upper panel) and t = 17 h (lower panel), (**B**) **2**, at t = 0 (upper panel) and t = 17 h (lower panel), (**C**) **3**, at t = 0 (upper panel) and t = 17 h (lower panel). In panel A, the m/z range between 1500 and 1900 is 10 times magnified.

ESI-MS spectra of NPM1_264–277_ in the presence of **1** and **2**, just upon the addition of the metal compound and after 17 h of incubation are reported in Figure 5. Immediately after the addition of **1**, ESI-MS spectrum of NPM1_264–277_ (Figure 5A, upper panel) indicates two predominant species with molecular weights of 2278.81 and 4561.57 Da, corresponding to NPM1_264–277_ monomer and dimer bound to one and two fragments of the Pt(II) compounds, respectively. The fragments of the Pt(II) adducts correspond to species in which **1** has lost Cl ligands (Table 2). This result is expected, based on the observed reactivity of the compound with proteins [22,27]. The amount of dimeric form bound to two Pt containing fragments increases over time (Figure 5A, lower panel). The spectra also that the NPM1_264–277_ monomer can bind two Pt containing fragments of **1** at the same time, as confirmed by the presence of a species at MW = 2789.96 Da. The presence of NPM1_264–277_ dimer bound to a higher number of Pt-containing fragments (molecular weights 5143.11 Da and 5108.63 Da, respectively) is also detectable.

Considering the preference of Pt compounds for specific amino acid residues [40,41], the sequence of the peptide and the finding that the Pt fragment binds both monomeric and dimeric forms of NPM1_264–277,_ it can be surmised that the most probable Pt binding sites are the side chains of lysine residues in positions 4 and 10 and of Glu in position 2. These residues were already found as ligands of Pt complexes [40,42,43].

Following the incubation of NPM1_264–277_ with **2**, the main species obtained just after the addition of **2** is the adduct of NPM1_264–277_ with one Pd-containing fragment constituted by a molecule of **2** missing Cl ligands (molecular weight = 2294.11 Da) (Figure 5B, upper panel). This species decreases over time (Figure 5B, lower panel). Additionally, the presence of species with molecular weights of 2189.17 and 2611.21 Da suggests that NPM1_264–277_ monomer binds one or two molecules of the Pd(II) compounds that have lost all Cl ligands NPM1_264–277_ monomer and dimer also show the ability to bind naked Pd(II) ions, indeed, peaks at 3749.08 Da and 1976.95 Da are assigned to NPM1_264–277_ dimeric and monomeric adducts with two and one metal ions, respectively. All these findings suggest that the state of thiol, free or covalently modified, together with the nature of the metal, highly influences the capability to bind the peptide of the investigated compounds_._ The metal complex fragments that are found bound to the peptide agree with those obtained upon reaction of the compound with proteins and suggest the possibility that a bidentate mode of binding could occur [22,27]. The ESI-MS data collected on the adducts formed in the reaction of NPM1_264–277_ with **1** and **2** are also in agreement with previous observations indicating that the investigated Pd compound has higher reactivity with proteins than the analogous Pt compound [22,27].

Altogether ESI-MS spectra unambiguously demonstrate that **1** and **2** react with the NPM1_264–277_ peptide forming stable adducts with a similar fragment (the molecule that has lost the Cl ligands), although the Pd compound seems to be able to degrade upon reaction with the peptide. These findings suggest that the mechanism of action of the two metal compounds in the aggregation of NPM1_264–277_ could be based on a direct interaction of **1** and **2** with monomeric and/or with low molecular weight oligomers of the peptide. Indeed, the finding that the compounds are also able to disaggregate the preformed fibrillar assembly suggests that the binding of the compounds to a specific site drives the oligomer to monomer equilibrium towards the monomeric species.

It is also interesting to note that both Pt(II) and Pd(II) compounds affect the oxidation of the Cys residue in NPM1_264–277_. Indeed, in the presence of both complexes, the amount of monomeric and the Cys-adduct monomeric species decreases over time, whereas the signals associated with the dimer increase (Figure 5A,B).

To evaluate if the oxidation state of NPM1_264–277_ could have a role in the aggregation process of NPM1_264–277_ in presence of the two compounds, the effect of the Pd(II) compound has been evaluated in the presence of DTT as reducing agent. Data, reported in Figure S5, indicate a delay in the decrease of fluorescence signal suggesting a role of the oxidation state of NPM1_264–277_ during its self-recognition mechanism.

### 3.5. **3** Does Not Form Adducts with NPM1_264–277,_ But It Significantly Affects the Number of Oxidized Forms of NPM1_264-277_

The spectra of NPM1_264–277_ in the presence of **3** show specific features that are different from those observed in the spectra of the peptide collected in the presence of **1** and **2**. No traces of adducts of 3 with NPM1_264–277_ can be detected even at longer incubation times. Nevertheless, in presence of the Au(III) compound, the intensities of the signals related to NPM1_264–277_ dimer decrease over time, leading to an increase of monomer and of its different oxidized forms (Figure 5C). This finding is in line with data indicating that the gold(III) compound reduces upon interaction with proteins [39,44] and is in line with what observed when other gold(III) compounds react with proteins and peptides [26]. The observed behavior of **3** can be explained on the basis of the reduction of Au(III) to Au(I) with thiols/thioethers and with the subsequent dismutation of Au(I) to Au(III) and Au(0). These processes can be accompanied by the formation of RSO_3_H species starting from RSH and RSSR, as suggested by some of us [44] and other authors [45].

These results indicate that the observed effects in NPM1_264–277_ aggregation by **3** are not due to a direct interaction between the metal complex and the peptide but through an indirect process that could involve a redox process.

### 3.6. Interactions of the Metal Complexes with Aβ_21–40_

The ESI-MS spectra of Aβ_21–40_ in absence of metal complexes highlight a weakness of the peptide bonds under the experimental conditions used for this analysis. During spectra acquisition using standard source settings, the peptide undergoes a fragmentation process, as evidenced by the presence in the spectra of several b-series signals (See Figure S4).

**Figure 6:**
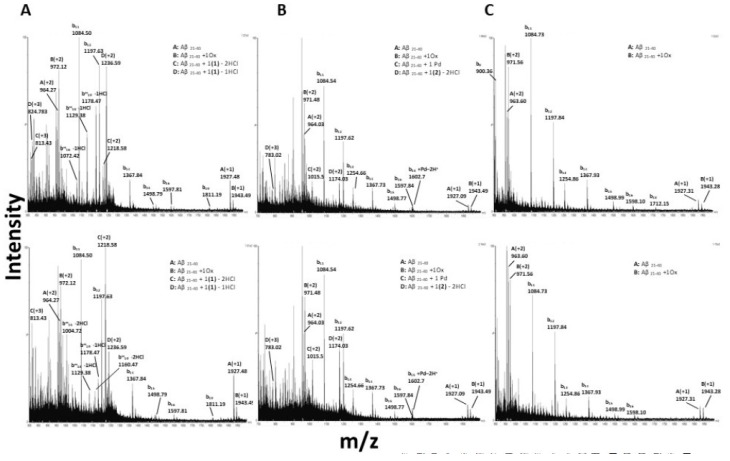
ESI-MS spectra of Aβ_21–40_ incubated with: (**A**) **1**, at t = 0 (upper panel) and t = 17 h (lower panel), (**B**) **2**, at t = 0 (upper panel) and t = 17 h (lower panel), (**C**) **3**, at t = 0 (upper panel) and t = 17 h (lower panel). In all panels, signals belonging to the fragmentation b series of Aβ_21–40_ are indicated. b series fragmentation signals deriving from Aβ_21–40_ complexed with metal compounds are indicated with an asterisk.

Just after the addition of **1** to Aβ_21–40_ (Figure 6A and Table 3), the peptide binds a Pt-containing fragment that has lost a Cl ligand, as indicated by the presence of the peak at 2471.54 Da. Over time, the peptide binds a Pt-containing fragment that has lost an additional Cl ligand (MW = 2435.59 Da).

When Aβ_21–40_ is incubated with **2** (Figure 6B), it immediately binds one molecule of the Pd(II) compound that has released two Cl ligands, as inferred from the species of 2346.03 Da molecular weight. Moreover, the presence of an adduct of 2029.00 ± 0.02 Da suggests that Aβ_21–40_ binds also a naked Pd(II) ion, as observed when the compound reacts with NPM1_264–277_. The adducts formed by Aβ_21–40_ with **1** and **2** are also present in the signals belonging to b-series. This finding suggests that the Aβ_21–40_ N-terminal tail is directly involved in metal compound recognition.

As observed in the reaction with NPM1_264–277_, **3** does not form adducts with Aβ_21–40_ (Figure 6C). However, the presence of the metal complex affects the oxidation state of the peptide. In particular, it promotes the oxidation of Met^35^, as demonstrated by a slight increasing of Aβ_21–40_ oxidized form over time (1941.68 Da).

These findings suggest that both the direct binding of metal complexes to the peptide and the oxidation of Met^35^ can be used as valuable strategies to inhibit the Aβ_21–40_ aggregation. These results are in line with previous data indicating that Met^35^ has a role in the aggregation process of Aβ_1–40_ [9,46]. To further corroborate this hypothesis, time-course ThT emission experiments on the oxidized Aβ_21–40_ were carried out (Figure S6). Data indicate that oxidized Aβ_21–40_ is unable to aggregate as its parent peptide Aβ_1–40_ [46].

## 4. Conclusions

**Figure 7:**
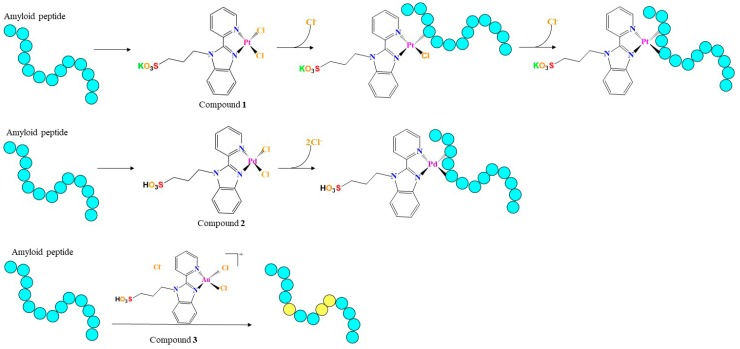
Schematic representation of the different mechanisms of action of compounds **1**, **2**, and **3** in the modulation of the aggregation of amyloid peptides analyzed in this study. Pt and Pd compounds interact with the peptides forming adducts with Pt- and Pd-containing fragments directly coordinated to residue side chains, while the Au compound does not directly interact with the peptides, but it alters the oxidation state of specific residues. In the figure, residues are indicated as cyan spheres. They are colored in yellow when present an altered oxidation state.

Pd(II)-, Pt(II)-, and Au(IIII)- complexes have been investigated for their ability to negatively modulate the aggregation of amyloid and amyloid-like peptides. However, a comparative study of the influence of the metal center on this inhibitory effect of the compound is missing. Here, the role of the metal center in the aggregation inhibitory activity of metal complexes bearing a benzimidazole ligand was investigated using analogues Au(III), Pt(II), and Pd(II) compounds. To the best of our knowledge this is the first paper reporting the effect of the metal center of analogous metal compounds on the aggregation of amyloid-like peptides. Two different sequences were investigated: the fragment corresponding to the 2nd helix of the C-terminal domain of nucleophosmin 1 (NPM1_264–277_), encompassing residues 264–277, which is able to form amyloid-like assemblies and fibrils toxic to neuroblastoma cells, and the fragment spanning residues 21–40 of the Aβ amyloid peptide (Aβ_21–40_). The investigated compounds significantly modulate the overall aggregation process of the peptides in vitro_,_ strongly suggesting an inhibitory action, even though only future morphological data deriving from electronic microscopy investigations can definitively assess the inhibition of the formation of amyloid fibers. In this respect, it should be noted that cytotoxicity experiments carried out using the investigated complexes have been reported to have CC50 values in the nanomolar range [22], while employed amyloid peptides, Aβ_21–40_ and NPM1_264–277_, showed cytotoxicity at high micromolar concentrations. These findings hamper the possibility to observe inhibition of toxicity of amyloid aggregates in presence of metal complexes. Thus, only future studies carried on different full-length amyloid proteins could confirm the anti-aggregation properties of this class of complexes and its potential therapeutic application in neurodegenerative diseases. On the other hand, our data provide interesting information on the peptide/metal compound recognition process. In detail the Pt and the Pd compounds interact with the peptides forming adducts with Pt- and Pd-containing fragments directly coordinated to residue side chains, while the Au compound does not directly interact with the peptides, although it significantly alters their redox state (as depicted in Figure 7). These data indicate that the compounds can use different mechanisms of action in the aggregation process of the peptides_._ This suggests that the modulation of aggregation process of amyloid peptide by metal compounds depends on a lot of factors that include, but are not limited to, oxidation state of metal, redox potential, stability, strength of metal-bond, total charge of the complex.

## Figures and Tables

**Table 1:** Peptide sequences analyzed in this study.

Peptide	Sequence	pI	Net Charge at pH7
NPM1_264–277_	VEAKFIN**Y**VKNCFR	10.15	1.9
Aβ_21–40_	AEDVGSNKGAIIGLMVGGVV	3.93	−1

**Table 2:** Results of the ESI-MS analysis of the adducts formed upon reaction of NPM1_264–277_ with **1**–**3**. The experimental m/z values, the ion charge status, the experimental (Exp) and theoretical (Theor) monoisotopic mass values, the corresponding ion species and times of incubation are reported.

Complex	Signal (m/z)	Charged Species	Exp MW (Da)	Theor MW (Da)	Species	Time (h)
1	1772.02 886.50	A (+1) A (+2)	1770.99 ± 0.02	1770.91	Monomer (NPM1_264–277_(M))	0,3,17
	1875.03 938.00 625.68	B (+1) B (+2) B (+3)	1874.02 ± 0.02	1873.91	Monomer Adduct (NPM1_264–277_(M_add_))	0,3,17
	1771.02 1181.33 885.99 709.00	C (+2) C (+3) C (+4) C (+5)	3540.22 ± 0.43	3539.82	Dimer (NPM1_264–277_(D))	0,3,17
	1140.539 760.360	D (+2) D (+3)	2278.81 ± 0.52	2281.45	NPM1_264–277_(M) + (1)−2HCl	0,3,17
	1396.095 931.070	E (+2) E (+3)	2789.960 ± 0.31	2791.9	NPM1_264–277_(M) + 2(1)−4HCl	0,3,17
						3,17
	1404.60 936.73	F (+2) F (+3)	2806.96 ± 0.30	2807.9	NPM1_264–277_(M) + 2(1)−4HCl + 1Ox †	0,3,17
						3,17
	1413.099 942.72	G (+2) G (+3)	2824.98 ± 0.60	2823.9	NPM1_264–277_(M) + 2(1)−4HCl + 2Ox	0,3,17
	1521.75 1141.06	H (+2) H (+4)	4561.57 ± 1.00	4560.9	NPM1_264–277_(D) +2(1)−4HCl	0,3,17
	1704.337 1278.03	I (+3) I (+4)	5108.63 ± 0.50	5107.8	NPM1_264-277_(D) + 3(1)−5HCl	0,3
						0,3,17
	1715.37	J (+3)	5143.11 *	5144.25	NPM1_264–277_(D) + 3(1)−4HCl	0,3
2	1772.146 886.560	A (+1) A (+2)	1771.12 ± 0.02	1770.91	Monomer (NPM1_264–277_(M))	0,3,17
	1875.100 938.060 625.720	B (+1) B (+2) B (+3)	1874.11 ± 0.02	1873.91	Monomer Adduct (NPM1_264–277_(M_add_))	0,3,17
	1771.14 1181.07 886.06 709.25	C (+2) C (+3) C (+4) C (+5)	3540.47 ± 0.44	3539.82	Dimer (NPM1_264–277_(D))	0,3,17
	1148.026 765.69	D (+2) D (+3)	2294.11 ± 0.09	2295.96	NPM1_264–277_(M_add_) + 1(2)−2HCl	0,3,17
	989.476	E (+2)	1976.952 *	1976.32	NPM1_264–277_(M_add_) + 1Pd	0,3,17
	1095.587	F (+2)	2189.17 *	2192.79	NPM1_264–277_(M) + 1(2)−2HCl	0,3,17
	1306.606	G (+2)	2611.21 *	2614.58	NPM1_264–277_(M) + 2(2)-4HCl	0,3,17
	1201.721	H (+3)	3602.19 ± 0.04	3604.00	NPM1_264–277_(D) + 4Ox	3,17
	1250.694	I (+3)	3749.082 *	3748.84	NPM1_264–277_(D) + 2Pd	0,3,17
3	1772.03 886.51	A (+1) A (+2)	1771.02 ± 0.01	1770.91	Monomer (NPM1_264–277_(M))	0,3,17
	938.00 625.68	B (+2) B (+3)	1874.98 ± 0.05	1873.91	Monomer Adduct (NPM1_264–277_(M_add_))	0,3,17
	1771.52 1181.01 886.01	C (+2) C (+3) C (+4)	3540.35 ± 0.48	3539.82	Dimer (NPM1_264–277_(D))	0,3,17
	893.51	D (+2)	1785.02 *	1786.91	NPM1_264–277_(M) +1Ox	0,3,17
	902.50	E (+2)	1803.00 *	1802.91	NPM1_264–277_(M) +2Ox	0,3,17
	910.51	F (+2)	1819.02 *	1818.91	NPM1_264–277_(M) +3Ox	0,3,17

* Error not available. † Ox refers to the presence of oxygen atoms on cysteine residues.

**Table 3:** Results of the ESI-MS analysis of the adducts formed by the reaction of Aβ_21–40_ with **1**, **2** and **3**. The experimental m/z values, the ion charge status, the experimental (Exp) and theoretical (Theor) monoisotopic mass values, the corresponding ion species and times of incubation are reported.

Complex	Signal (m/z)	Charged Species	Exp MW (Da)	Theor MW (Da)	Species	Time (h)
1	1927.48 964.27	A (+1) A (+2)	1926.50 ± 0.02	1926.00	Aβ_21–40_	0,3,17
	1943.49 972.12	B (+1) B (+2)	1942.35 ± 0.13	1942.00	Aβ_21–40_+ 1Ox ^†^	0,3,17
	1218.583 813.429	C (+2) C (+3)	2435.59 ± 0.56	2436.45	Aβ_21–40_ + 1(1)-2HCl	0,3,17
	1236.599 824.641	D (+2) D (+3)	2471.54 ± 0.36	2472.9	Aβ_21–40_ + 1(1)-1HCl	0,3,17
						0,3
2	1927.09 964.03	A (+1) A (+2)	1926.07 ± 0.02	1926.00	Aβ_21–40_	0,3,17
	1943.07 971.48	B (+1) B (+2)	1941.50 ± 0.56	1942.00	Aβ_21–40_ + 1Ox	0,3,17
	2030.029 1015.500	C (+1) C (+2)	2029.00 ± 0.02	2028.42	Aβ_21–40_ + 1 Pd	0,3,17
	1174.035 783.022	D (+2) D (+3)	2346.03 ± 0.02	2347.79	Aβ_21–40_ + 1(2)-2HCl	0,3,17
3	1927.31 963.60	A (+1) A (+2)	1925.74 ± 0.56	1926.00	Aβ_21–40_	0,3,17
	1943.28 971.56	B (+1) B (+2)	1941.68 ± 0.58	1942.00	Aβ_21–40_ +1Ox	0,3,17

† Ox refers to the presence of an oxygen atom on the methionine residue.

## Supplementary Materials

The following are available online at https://www.mdpi.com/1424-8247/12/4/154/s1, Figure S1: UV-Vis absorption spectra of **1** (panel A), **2** (panel B), and **3** (panel C) over 24 h under the following experimental conditions: 10 mM ammonium acetate buffer pH 6.8, 10 mM sodium phosphate buffer pH 7.4 and 10 mM borate buffer pH 9.0. Spectra have been acquired at a compound concentration of 5*10^−5^ M. Final concentration of DMSO is 0.5%; Figure S2: Absorption spectra of NPM1_264–277_ at increasing concetrations (indicated by arrow) that correspond to those added during the titration in presence of **1**, **2** and **3**, reported in Figure 5 upper panel. (0 µM fuchsia, 20 µM black, 40 µM orange, 60 µM blue, 80 µM violet, 100 µM yellow, 120 µM light blue, 140 µM green, 160 µM grey); Figure S3: ESI-MS spectra of NPM1_264–277_ alone in the absence (panel A) and in the presence (panel B) of DTT. Component A: monomer (MM = 1771.17 + 0.16 Da); component B: adduct (MM = 1873.98 + 0.05 Da); component C: dimer (MM = 3540.01 + 0.01 Da); Figure S4: ESI-MS spectrum of Aβ_21–40_ peptide alone. Component A: Aβ_21–40_ (MM = 1926.24 + 0.05 Da); B:Met oxidized Aβ_21-40_: (MM = 1941.67 + 0.60 Da). Aβ_21–40_ fragments belonging to b-series and spontaneously generated in source are also present; Figure S5. Time course of ThT fluorescence emission intensity of NPM1_264–277_ in presence of **2** and DTT (10 mM) as reducing agent; Figure S6: Time course of ThT fluorescence emission intensity of Aβ_21–40_ in reduced and oxidized forms.
