# Supplementary material for: Role of the Metal Center in the Modulation of the Aggregation Process of Amyloid Model Systems by Square Planar Complexes Bearing 2-(2′-pyridyl)benzimidazole Ligands
Source: Pharmaceuticals (Basel). 2019 Oct 12;12(4):154. doi: 10.3390/ph12040154 (PMC6958441; doi:10.3390/ph12040154)
Supplement: Supplementary file 1 [file pharmaceuticals-12-00154-s001.pdf]

Article

Supporting info

Role of the metal center in the inhibition of the aggregation process of amyloid model systems by square planar complexes bearing 2-(2'-pyridyl)benzimidazole ligands

Daniele Florio [1,^], Ilaria Iacobucci [2, 3,^], Giarita Ferraro[4], Ahmed M. Mansour[5], Giancarlo Morelli [1] Maria Monti [2,3,] Antonello Merlino[2,*] and Daniela Marasco[1,*]

[1]Department of Pharmacy, University of Naples Federico II, Napoli, Italy; floriodaniele1@gmail.com, daniela.marasco@unina.it

[2]Department of Chemical Sciences, University of Naples Federico II, Napoli, Italy; ilaria.iacobucci@unina.it, montimar@unina.it, antonello.merlino@unina.it

[3] CEINGE Biotecnologie Avanzate S.c.a r.l., University of Naples Federico II, Napoli, Italy

[4]Department of Chemistry Ugo Schiff, University of Florence, Sesto Fiorentino (FI), Italy giarita.ferraro@gmail.com,

[5]Department of Chemistry, University of Cairo, Egypt. mansour@sci.cu.edu.eg

*Correspondence: antonello.merlino@unina.it or daniela.marasco@unina.it; Tel.: +39081674276 A.M or +390812534607 D.M.

*Structural stability of **1**, **2** and **3***

UV-Vis absorption spectroscopy was used to study the in solution stability of the three compounds under the conditions that were needed to perform the experiments described in this work. Figure S1 reports the UV-Vis spectra of **1**, **2** and **3** in 10 mM ammonium acetate buffer pH 6.8, sodium phosphate buffer pH 7.4 and borate buffer pH 9.0 as function of time. The UV-Vis spectra of **1** show a single absorption peak at 285 nm, while those of **1** and **2** present two peaks, one at 268 nm and one more intense at 332 nm, which can be attributed to Ligand to Metal or Metal to Ligand Charge Transfer (LMCT or MLCT).

The spectral profiles of **2** and **3** remain unchanged over 24 h, suggesting that they are stable under the analyzed experimental conditions. This indicates that these two compounds do not undergo ligand exchange or hydrolysis reaction processes within 24 h. On the contrary, **1** has a slightly different behavior when compared to its analogues. In particular, in sodium phosphate and sodium acetate buffer, it experiences an increase of the absorption which could be related to modifications of its ligand field and a small shift of maximum absorption wavelength (λ_{max}) from 286 to 289 nm.

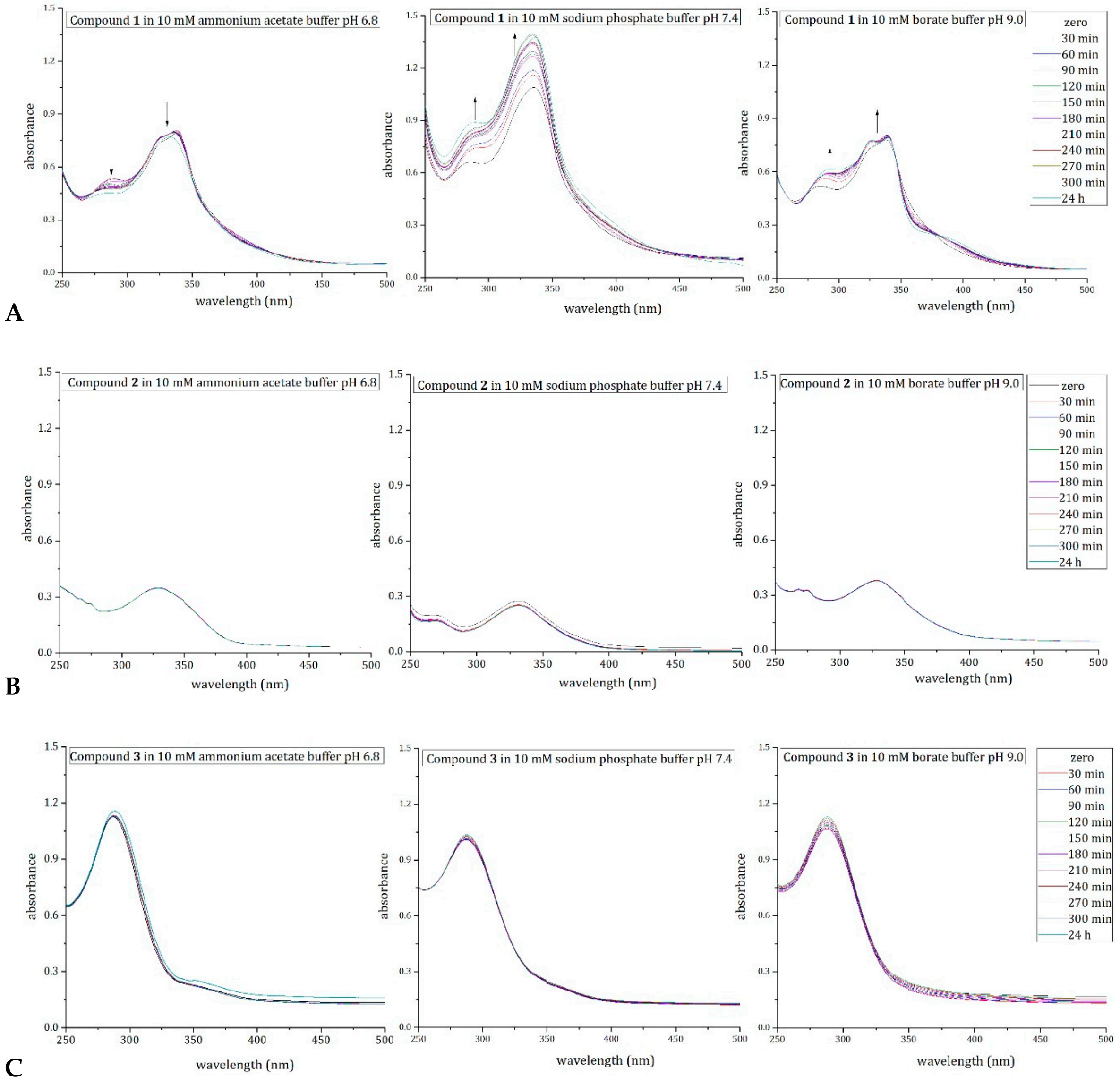

Figure S1: UV-Vis absorption spectra of **1** (panel A), **2** (panel B), and **3** (panel C) over 24 h under the following experimental conditions: 10 mM ammonium acetate buffer pH 6.8, 10 mM sodium phosphate buffer pH 7.4 and 10 mM borate buffer pH 9.0. Spectra have been acquired at a compound concentration of $5*10^{-5}$ M. Final concentration of DMSO is 0.5%.

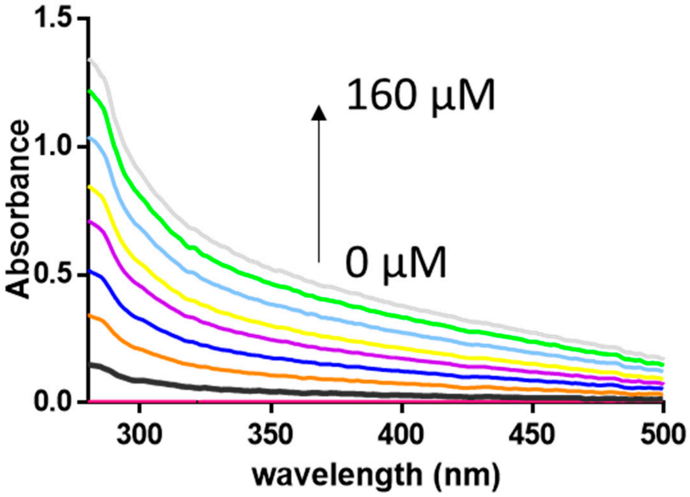

Figure S2: Absorption spectra of NPM1$_{264\text{-}277}$ at increasing concetrations (indicated by arrow) that correspond to those added during the titration in presence of **1, 2** and **3** reported in Figure 5 upper panel. (0 µM fuchsia, 20 µM black, 40 µM orange, 60 µM blu, 80 µM violet, 100 µM yellow, 120 µM ligth blu, 140 µM green, 160 µM grey)

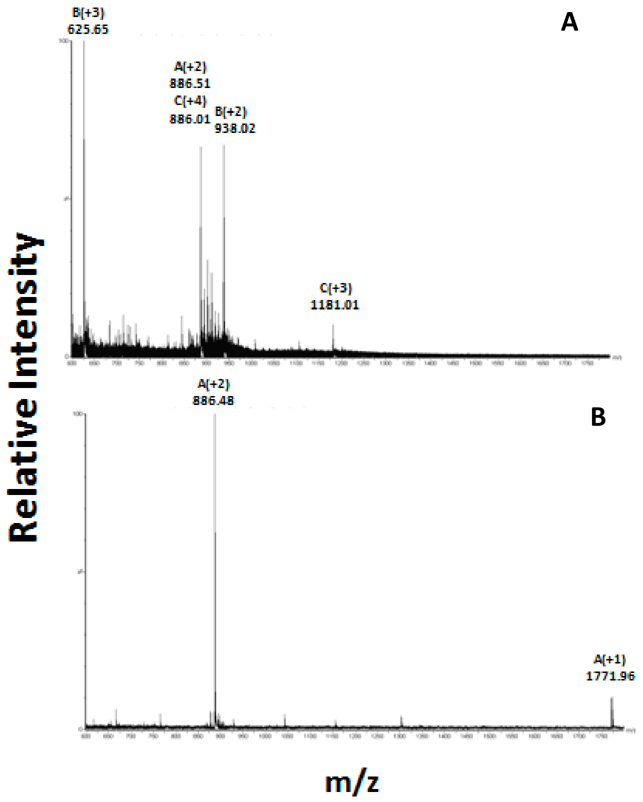

Figure S3: ESI-MS spectra of NPM1$_{264-277}$ alone in the absence (panel A) and in the presence (panel B) of DTT. Component A: monomer (MM=1771.17$\pm$0.16 Da); component B: adduct (MM=1873.98$\pm$0.05 Da); component C: dimer (MM=3540.01$\pm$0.01 Da).

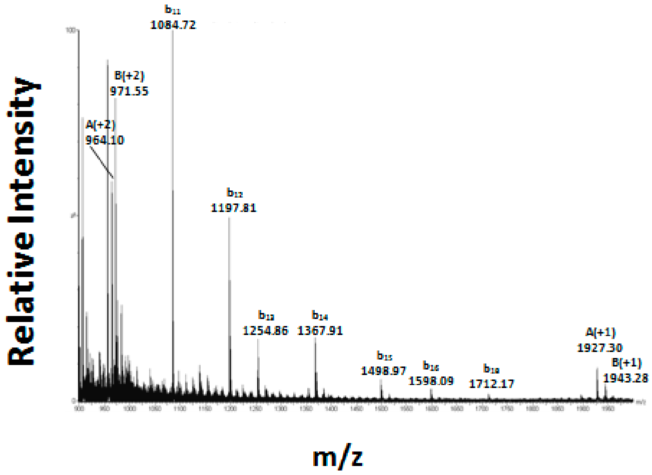

Figure S4: ESI-MS spectrum of Aβ_{21-40} peptide alone. Component A: Aβ_{21-40} (MM=1926.24$\pm$0.05 Da); B:Met oxidized Aβ_{21-40}: (MM=1941.67$\pm$0.60 Da). Aβ_{21-40} fragments belonging to b-series and spontaneously generated in source are also present.

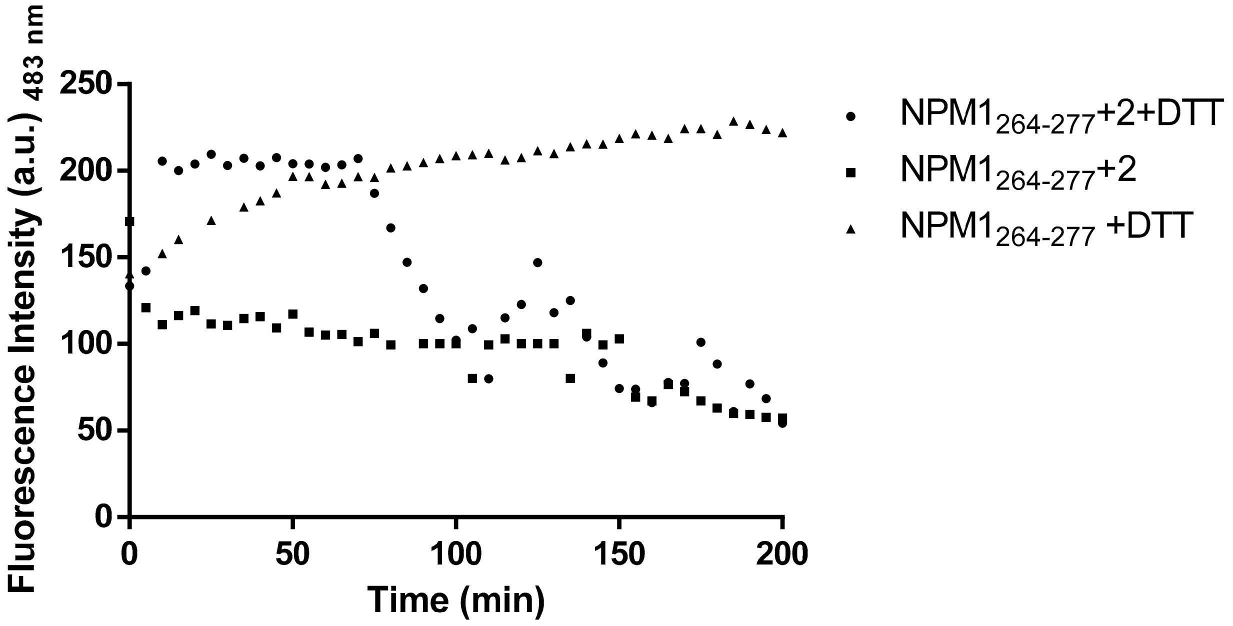

Figure S5. Time course of ThT fluorescence emission intensity of NPM1$_{264-277}$ in presence of **2** and DTT (10 mM) as reducing agent.

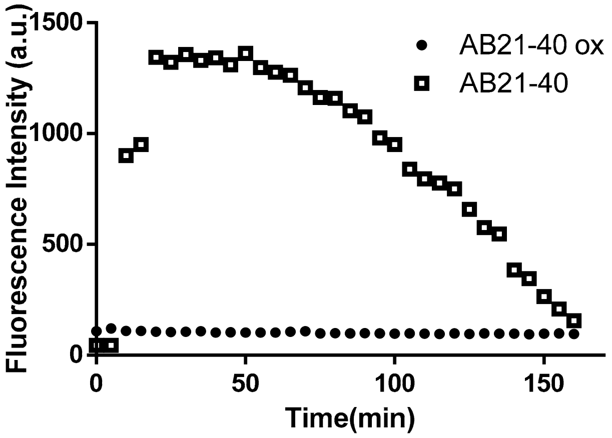

Figure S6: Time course of ThT fluorescence emission intensity of Aβ $_{21-40}$ in reduced and oxidized forms.
